# The Functional Dilemma of Nectar Mimic Staminodes in *Parnassia wightiana* (Celastraceae): Attracting Pollinators and Florivorous Beetles

**DOI:** 10.1002/ece3.70380

**Published:** 2024-09-30

**Authors:** Shi‐Jia Wen, Shan Chen, Andre Rodrigo Rech, Ling Ji, Hong Wang, Zhiyong Wang, Ding Wu, Zong‐Xin Ren

**Affiliations:** ^1^ Key Laboratory for Plant Diversity and Biogeography of East Asia Kunming Institute of Botany, Chinese Academy of Sciences Kunming China; ^2^ College of Forestry Hainan University Haikou China; ^3^ Jiangxi Key Laboratory of Plant Resources and Biodiversity Jingdezhen University Jingdezhen China; ^4^ Shanghai Pudong Zhengda Foreign Language School Shanghai China; ^5^ Universidade Federal dos Vales do Jequitinhonha e Mucuri Diamantina Minas Gerais Brazil; ^6^ College of Forestry Jiangxi Agricultural University Nanchang China; ^7^ Lijiang Forest Biodiversity National Observation and Research Station Lijiang China

**Keywords:** floral trait, florivory, nectary‐mimicking staminode, pollinator attraction, reproductive success, staminodes

## Abstract

While floral signaling plays a central role in the reproductive success of all animal‐pollinated plants, it may also attract herbivores eager to feed on flowers. False nectaries with glossy surfaces reflecting incident light may produce signals that attract floral visitors guiding their movements to and within the flower. Whether false nectaries also attract herbivores that lower the reproductive success of natural populations requires attention. In this study, we focus on *Parnassia wightiana*, a subalpine species with a whorl of staminodes that act as false nectaries attracting bees, flies, and herbivorous beetles. We tested the functions of staminodes using controlled manipulative experiments under field and lab conditions. We found a significant decrease in pollinator visits, and subsequent seed set, in flowers in which we removed staminodes or staminode apices confirming the function of these organs. In our natural populations, we found that a beetle, *Nonarthra variabilis* (Alticinae; Chrysomelidae), chews first on staminode apices, then it eats the entire staminodes and other flower parts, but rarely feeds on ovaries. Additional experiments suggested these beetles preferred staminodes to ovaries. Our results suggest this is a case of selective florivory, in which staminodes play a dual role, attracting pollinators and herbivores at the same time causing the attractive dilemma. Although selective florivory by beetles did not directly damage fruits, it influenced plant‐pollinator interactions, decreasing reproductive success in plant populations. Our study highlights the importance of plant‐pollinator‐herbivore interactions in selecting floral traits.

## Graphical abstract

Staminodes of *Parnassia wightiana* acting as false nectaries attract both pollinators and florivorous beetles, and subsequently influence plant reproductive success.

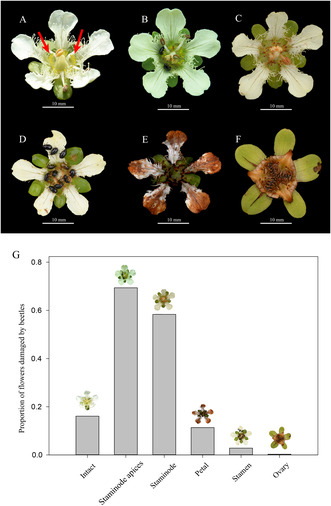

## 1 Introduction

Visually oriented animals are required to ensure pollination and subsequent fruit set in most flowering plants (Brito, Telles, and Lunau 2014; Ollerton, 2011). Consequently, most flowers rely on a combination of visual stimuli to advertise their presence, reinforced with limited supplies of rewards consumed and/or gathered directly and regularly by their pollinators (Chittka and Menzel 1992). By increasing their detectability, zoophilous flowers face an attractive dilemma. The same signals and rewards that attract pollinators may also attract herbivores, while floral traits deterring florivores may also deter pollinators (Mccall and Irwin 2006; Campbell et al. 2022). For example, the larger and longer‐lived flowers of *Cistus ladanifer* L. (Cistaceae) receive more pollinator visitations increasing their reproductive success, but the same flowers also tend to be damaged more often by florivorous beetles (Teixido, Méndez, and Valladares 2011). Florivores can strongly influence a plant's reproductive success in more than one way. They may cause direct damage to pollen grains and ovules and/or to floral organs that emit scents that also guide pollinators (Theis and Adler 2012). Their consumption changes floral morphology and flower number (Strauss 1997) on the same plant reducing visitation rates of pollinators (Penet, Collin, and Ashman 2009; Sõber, Moora, and Teder 2010). An ideal solution to solve the attractive dilemma would be a floral strategy that attracts pollinators at the same time it becomes unattractive to all its antagonists including florivores and reward robbers (Lunau et al. 2011). A second possibility would be to select for a mode of floral presentation that would incur damage to some sterile organs while deflecting florivores away from the reproductive organs (Maldonado et al. 2015; Bernhardt 2000). Unfortunately, the extent of the effect of florivory and anti‐florivore defense on floral trait evolution, plant‐pollinator interaction, and plant reproduction remains understudied (Irwin, Adler, and Brody 2004).

We also understand that the consumption of nectar is one of the most common reasons why prospective pollinators forage on flowers. However, with important exceptions (Zhang et al. 2012), the standard model as expressed by pollination ecologists is that, in general, floral nectar appears hidden within flowers or does not appear to be conspicuous immediately to prospective pollinators. This truism is changing thanks, in part, due to recent studies by Lunau et al. (2020) who apply false color photography (Verhoeven, Ren, and Lunau 2018) to address nectar mimicry. They found that some nectar secretions, and/or whole nectar glands and false nectaries are often located in centralized UV‐absorbing parts of the flower. These parts have glossy surfaces reflecting all incident light and produce strong UV‐signals and they are highly visible to UV‐sensitive animals. Nectar mimic signals should attract floral visitors and guide their movements toward true nectar rewards (Liao et al. 2020). In particular, Lunau et al. (2020) found that some herbs growing at subalpine elevations on the Hengduan Mountains showed nectar mimicry. They either flaunted glossy protuberances such as on their petals (e.g., *Saxifraga* spp.) or produced a whorl staminodes with glossy, swollen apices, as in *Parnassia wightiana* Wallich ex Wight & Arnott (Celastraceae; Figure 1j–l in Lunau et al. 2020). While the glossy staminodes of European *P. palustris* L. have long been interpreted as false nectaries attracting fly pollinators (Daumann 1960; Kugler 1951), we lack experimental evidence to consider staminode function in its congeners. Likewise, we do not know whether the shiny staminodes in *Parnassia* species also attract florivores that could lower fecundity in natural populations.

**FIGURE 1:**
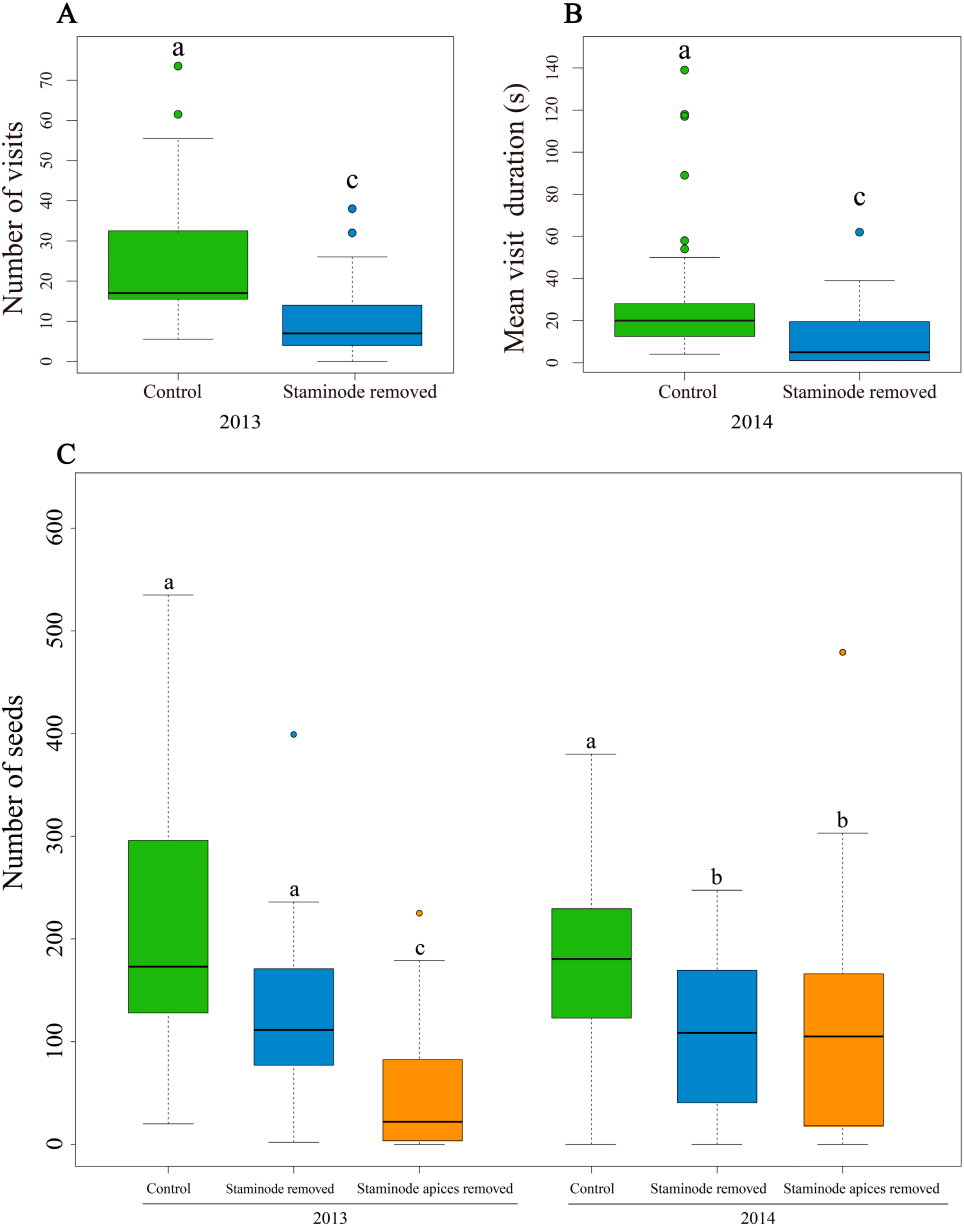
The effect of the removal of staminode and staminode apices on plant reproduction. (A) Number of potential pollinating visitors; (B) the mean durations of visits to treated flowers; (C) number of seeds per fruit. Different lowercase letters indicate significant differences between treatments (a vs. b indicates *p* < 0.01; a vs. c indicates *p* < 0.001).

In this study, we chose *P. wightiana* as a model species for its staminodes to test the attractive dilemma. The visual character of the staminode apices was defined by Lunau et al. (2020) because they reflected UV light. Previous studies suggest it is a generalist pollinated species, visited by a combination of bees, flies, and beetles (Armbruster et al. 2014). Li, Ren, and Li (2021) collected and identified flower‐visiting beetles systematically on Yulong Snow Mountain. They found that flowers of *P. wightiana* are visited by 10 species of florivorous beetles and *Nonarthra variabilis* (Alticinae; Chrysomelidae) dominates. These beetles feed on staminodes, stamens, and petals. Therefore, the staminodes of *P. wightiana* may pose as an attractive dilemma.

By conducting field observations and controlled manipulative experiments, we address the following questions. Do false nectar structures (staminodes) attract floral visitors? Does removal of staminodes decrease the abundance and duration of floral visitation? If this is the case, does the removal of staminodes result in a decrease in seed production in wild populations? What is the level of damage imposed by known florivorous beetles to plant populations? Specifically, does beetle florivory decrease seed production? We proposed two hypotheses: (1) staminodes attract pollinators but also attract florivores; (2) florivorous beetles damage staminodes but not ovules. Instead, the consumption of staminodes by beetles lowers reproductive success, because it deters pollinator visitation during the floral lifespan.

## 2 Materials and Methods

### 2.1 Study Species and Sites

*Parnassia wightiana* is a perennial, geophytic herb distributed in southern China and south into northern Thailand, then west to southwestern China, and northern India, Nepal, and Bhutan. It is found usually in open woodlands, grassy pastures, and along roadsides at elevations from 600 to 3400 m. At higher elevations, this species grows in sub‐alpine and alpine wet meadows. Each mature plant of *P. wightiana* produces a single flower with a long erect flowering stem each year. Individual flowers are large at 29.61 ± 3.16 mm (mean ± SD, *n* = 30) in diameter. The corolla is white to the human eye. Each flower contains a whorl of five staminodes. Each staminode terminates into 5‐lobes, occasionally bearing an inconspicuous nectar‐like glandular dot at its apex. The true floral nectary is located at the base of each staminode. Nectar secretions are visible to the human but each individual gland appears to secrete < 1 μL of fluid. The flower is protandrous showing a stamen cascade movement (Armbruster et al. 2014; Ren and Bu 2014). The floral lifespan of *P. wightiana* is > 10 days. The male phase lasts > 5 days followed by the slow opening and spreading of the trifid‐lobed stigma. The stigmatic surfaces appear to remain receptive for more than 5 days. Spontaneous mechanical self‐pollination was not observed in this species (S. Chen, unpublished data). Insects in the Orders Diptera (Tephritidae, Syrphidae, Muscidae, Dolichopodidae, Anthomyiidae, and Empididae) and Hymenoptera (Tenthredinidae, Formicidae, and Apidae) visit flowers for nectar and/or pollen, predation (i.e. spiders), rest and mating (S. Chen, unpublished data).

Our observations and experiments were conducted in the Lijiang Forest Biodiversity National Observation and Research Station located at 3300 m on Yulong Snow Mountain. We selected three study sites with two sites at a high elevation and one site at a lower elevation. Site A (26°59′35.7″ N, 100°10′32.8″ E, 3303 m) and site B (26°59′46.9″  N, 100°10′ 20.2″ E, 3308 m) are paired sites separated by 1.5 km. Site A population contained > 500 flowering individuals with an abundance of beetles feeding on their flowers. Site B contained about 300 flowering plants and appeared less infested by beetles. Both sites were meadows in the pine tree forest, used intensely for pollination studies since 2012 (Li, Ren, and Li 2021; Liang et al. 2021; Xu et al. 2021). The lower elevation Site C (Yushuizhai, 26°59′57.9″ N, 100°11′55.7″ E, 2721 m) contained > 200 flowering plants without of beetle infestation. This site was used for a pollination network study by Zhao et al. (2019). In all three sites, plants of *P. wightiana* were distributed in irregular clumps of patches of 2–20 flowering plants. Flowering occurred from June to September, with our two, high elevation populations flowering first, about 20 days earlier than the low‐elevation population. Fruits matured in September. Two other species in the genus *Parnassia*, *P. delavayi* Franchet, and *P. mysorensis* F. Heyne ex Wight & Arnott were co‐occurring and co‐blooming at our two, high elevation sites, but all three species occupied different microhabitats. *P wightiana* grew exposed in wet meadows, *P. delavayi* under trees, and *P. mysorensis* in drier, upper grassland slopes. We also used flowers of the two latter species to perform feeding choice experiments (see below) to test if beetles feed on their floral parts too.

### 2.2 Staminodes Removal Experiments

To test the function of staminodes and of the glossy staminodes apices, we performed a series of paired manipulation experiments to compare the effects of the removal of the entire staminode versus only its apices on visiting pollinators to compare the number of visits, visitation duration by insects and seed production (number of seeds/fruit). In 2023, we chose 80 plants (40 pairs) in buds in site B for manipulative experiments. In Site A, we found that beetles frequently chewed peduncles of flowers we bagged and/or tagged (Figure S1), such behavior caused damage of plants influencing manipulating experiments. Therefore, we chose Site B because it showed less damage to flowers, beetles were less likely to influence the experiment. Each of the paired plants were separated from each other by at least 10 cm. When flowers were fully open on one of the two, paired plants we used a scissor to cut off its five staminodes at their bases and then marked the peduncle with purple yarn. The flower on the second plant was left intact and marked with red yarn. To compare visitation rates, we observed and counted the number of floral visitors landing on each flower on each pair six times over their respective flowering periods. Each observation period lasted 10 min. Therefore, in total, each pair received 60 min of observation. Only insects, with body parts large enough to contact dehiscent anthers or receptive stigmas while foraging were recorded as potential pollinators. In 2014, we did not count the number of visits, but recorded the time (in seconds) each insect spent on an individual flower (with and without staminodes). In total, we recorded a duration of 47 insect visits to control flowers, and 27 visits to flowers with staminodes removed. The flowers of both plants were then checked for fruit and seed set in September (see below).

We also tested the effect of removing staminode apices versus the entire staminodes on the seed production. We selected at random 170 flowers and tagged them. We used scissors to either remove the entire staminodes, or we only snipped off their apices (upper 1 mm of each staminode). Controls were not cut. As mature capsules dehisce to disperse their seeds, we bagged all flowers used in this experiment with green mesh organza bags as petals withered at the end of flowering season. In September, we collected all capsules. As some tagged specimens were damaged by cattle we collected a total of 64 control fruits, 30 fruits from flowers in which all staminodes were removed, and 44 fruits in which staminode apices were removed. The same experiment was repeated in 2014 resulting in 40, 36, and 42 fruits for control, entire staminode removal, and apice removal experiment, respectively. Seeds of each bagged fruit were counted in the lab.

### 2.3 Florivory Dynamics

To describe foraging of beetle florivores on *P. wightiana*, we began our observations after July 10, 2014 on Site A where beetles were found most frequently on flowers. While flowers began blooming in this population, beetles did not begin feeding in them until July. Beetles were most abundant on flowers after July 10 until early August as flowers began to wither. We recorded which floral organs the beetles fed upon and which organs they moved to sequentially after their initial grazing on the staminodes. We tagged 100 flowers at random and then counted the number of flowers with structures damaged by beetles: staminode apices, whole staminodes, petals, anthers, and ovary. Beetle specimens were collected and stored in centrifuge tubes in the field, dried with silica gel, and then sent to an entomologist for identification. Specimens are deposited in the Kunming Institute of Botany, Chinese Academy of Sciences.

### 2.4 Feeding Choice Experiments

During field observations, we found that beetles first chewed on staminodes. We, therefore, wondered whether they preferred staminodes to other floral structures. In 2022, we conducted Petri dish arena choice experiments following the methods of Netherer et al. (2022). We conducted 405 experimental trials. All experiments were conducted in the same room under white light.

**FIGURE 2:**
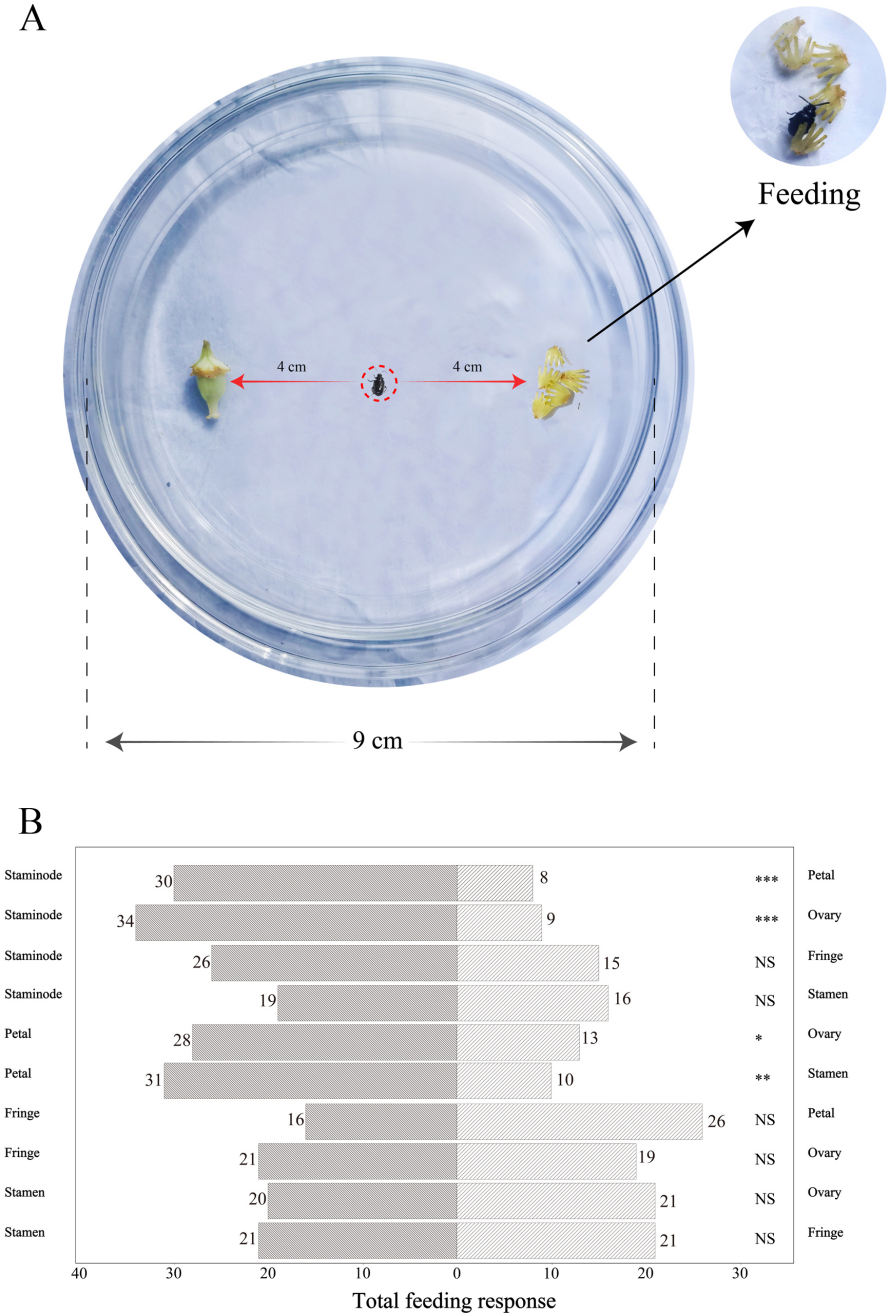
Petri dish arena choice experimental design (A) and results (B) preference of florivorous beetles for staminodes, stamens, and petals. NS *p* > 0.05, *p < 0.05, **p < 0.01, ***p < 0.001.

All choice trials were performed in glass Petri dishes (9 cm diameter). Flower parts were placed on a line at each side within a distance of 4 cm from the dish center (Figure 2A). The following flower parts were used for the one‐by‐one choice experiment: whole staminode, whole petal, petal marginal fringes, whole stamen, and ovary. All the flower parts were freshly collected from Site B and then brought back to the lab in the field station. All flower parts used for the experiment were carefully dissected from whole flowers before using forceps to transfer them to the Petri dish. Beetles used for experiments were collected from Site A 1 day before the choice experiment and kept in an empty Petri dish. They were unfed for 24 h before they were used in this choice experiment. One beetle was placed in the center of the Petri dish, and then, a transparent lid was placed over the dish. Each beetle was observed for a period of 10 min. We only counted the beetle's choice if it was observed feeding on a floral structure (Figure 2A). Mere movement towards a floral structure was not counted. The result of each choice trial was recorded as either 0 (no feeding) or 1 (feeding) for each flower part offered. In total, we had 10 choice experimental treatments with each treatment repeated 35–42 times. For each replicate, we used fresh, previously unused new floral parts and a previously unused and presumably hungry beetle.

We observed the same beetle species feeding on flowers of two congeners of *Parnassia*: *P. delavayi* and *P. mysorensis* and other co‐blooming species, including *Ligularia alatipes* Handel‐Mazzetti (Asteraceae), *Leontopodium calocephalum* (Franchet) Beauverd (Asteraceae), and *Saxifraga diversifolia* Wallich ex Seringe. To test if beetles showed the same grazing preferences in the flowers of the two *Parnassia* congeners, we used staminodes and other floral parts of the other two species to do the same feeding choice experiments using the same experimental design as for *P. wightiana*. Using the same experimental design we also tested to determine whether beetles preferred the staminodes of *P. wightiana* over those of stamens of other co‐blooming flowering plants.

### 2.5 Reproductive Success Among Populations

We compared reproductive success of plants in our three sites with different level of beetle infestation. In Site A, we initially marked 35 flowers infested by beetles and 35 flowers without infestation at the male stage. However, beetles always chewed peduncles of marked flowers (see Figure S1) and also chewed floral parts for none infestation groups. In the end, more than half of marked flowers were damaged and all remaining flowers (31 flowers) were infested by beetles. Because of such strong damage by beetles, we cannot design an experiment by comparing flowers with or without infestation to test the effect of beetle infestation on plant reproductive success using population as replica. Therefore, we collected pistils to check for pollen tube growth as a proxy for reproductive success avoiding severe damage by beetles. Furthermore, we marked 50 intact (without beetle‐infested), male stage flowers in each site of B and C for comparison among populations. In the end, we were able to collect 31 pistils from flowers in Site A, and 33 and 42 pistils from flowers from Site B and Site C, respectively. All flowers from Site A were infested by beetles, and all flowers from Site B and C were more or less likely free of beetle infestation. All the collected pistils were at the female stage with petals wilt, with stigma surface exposing to insect visitation for 3 to 4 days. Pistils were fixed in 3:1 ethanol (0.95): glacial acetic acid for 2 h and then preserved in 70% ethanol. In the lab, we observed pollen tube growth and counted the number of pollen tubes/pistil under an epifluorescence microscope using an Axio Lab.A1 (Zeiss, Oberkochen, Germany). The fluorescence staining method and pollen tube observation follow Ren et al. (2019). Beetle florivory began during July in Site A and Site B (see above), and the flowering time at Site C began about 20 days later than at other two, higher sites. Therefore, we collected marked pistils in Site A, B on July 24, 2014, and marked pistils in Site C on August 10, 2014.

Additionally, we also compared seed production for plants during the early, middle and late flowering season at Site A. In Site A, we subdivided the flowering plants by their flowering time into three stages, early (flowering in June), middle (July 1–July 15) and late (July 16–August 1). This subdivision follows the increasing abundance of beetles on flowers from early (no beetles), middle (< 20% of flowers with chewing beetles), and late (> 70% of flowers with chewing beetles). We marked and bagged 60 flowers with wilting petals from each time stage for fruit collection, but some of the flowers were damaged by beetles by chewing the peduncle, we were only able to collect 31 (early), 41 (middle), and 25 (late) fruits in September. To quantify the seed set at each stage, the number of seeds/fruit was counted using the same method as above.

### 2.6 Statistical Analyses

All of the data from this study failed to pass the normality test (Shapiro–Wilk, *p* < 0.05). Therefore, we used the non‐parametric Mann–Whitney *U*‐test to compare insect visitation numbers and the floral visit duration on differently treated flowers between control and staminode removed treatment. The number of seeds per fruit for different treatments (control, whole staminode removal, and staminode apices removal) and flowering stages (early, middle, and late) was also compared using the Kruskal–Wallis test, a non‐parametric analysis of variance. The number of pollen tubes for flowers from different populations was compared by Kruskal–Wallis test too. If significant differences were detected, a Dunn's Method post hoc test was applied to determine the sources of differences. Statistical analyses were performed by SigmaPlot (SigmaPlot for Windows 2013; Systat Software Inc., Richmond, USA) and boxplot figures were drawn by R (version 4.2.2; R Core Team 2022).

We used exact binomial tests of goodness‐of‐fit to evaluate differences in feeding choices among floral organs. The tests were run using the spreadsheet provided by http://www.biostathandbook.com/exactbin.xls (access time: October 13, 2022). The null model hypothesis was that all floral parts used in the choice experiments attracted the same number of beetles.

## 3 Results

### 3.1 Staminode Removal Experiments

Compared with intact controls, flowers in which the ring of staminodes were removed received fewer insect visits (Mann–Whitney test: *U* = 57.045, df = 2, *p* < 0.001; Figure 1A), and the visits they received lasted shorter periods (*U* = 42.215, *p* < 0.001; Figure 1B). Floral visitation behavior also differed between staminode removed and intact flowers. When a floral visitor landed on an intact control flower, it moved around the whole whorl of staminodes to drink floral nectar at their bases. When staminode whole was absent insects landed on the flower without moving among the stamens or stigmas and they quickly flew off. We also observed that when an insect visited flowers with only staminode apices removed, it moved around the staminode whorl but then flew away without foraging at all the functional nectar glands.

The number of seeds per fruit was significantly different among flowers with staminodes removed, with staminode apices removed and control in 2013 (Kruskal–Wallis test: *χ* ^2^ = 46.973, df = 2, *p* < 0.001; Figure 1C) and 2014 (*χ* ^2^ = 14.303, df = 2, *p* < 0.001; Figure 1C). In both years, seed productions of flowers with staminode apices removed were significantly lower than control, intact flowers (both *p* < 0.001). The effect of complete staminode removal versus staminode apices removal also differed in 2013 (*p* < 0.001), but not in 2014 (*p* = 1.000).

### 3.2 Florivory Dynamics

**FIGURE 3:**
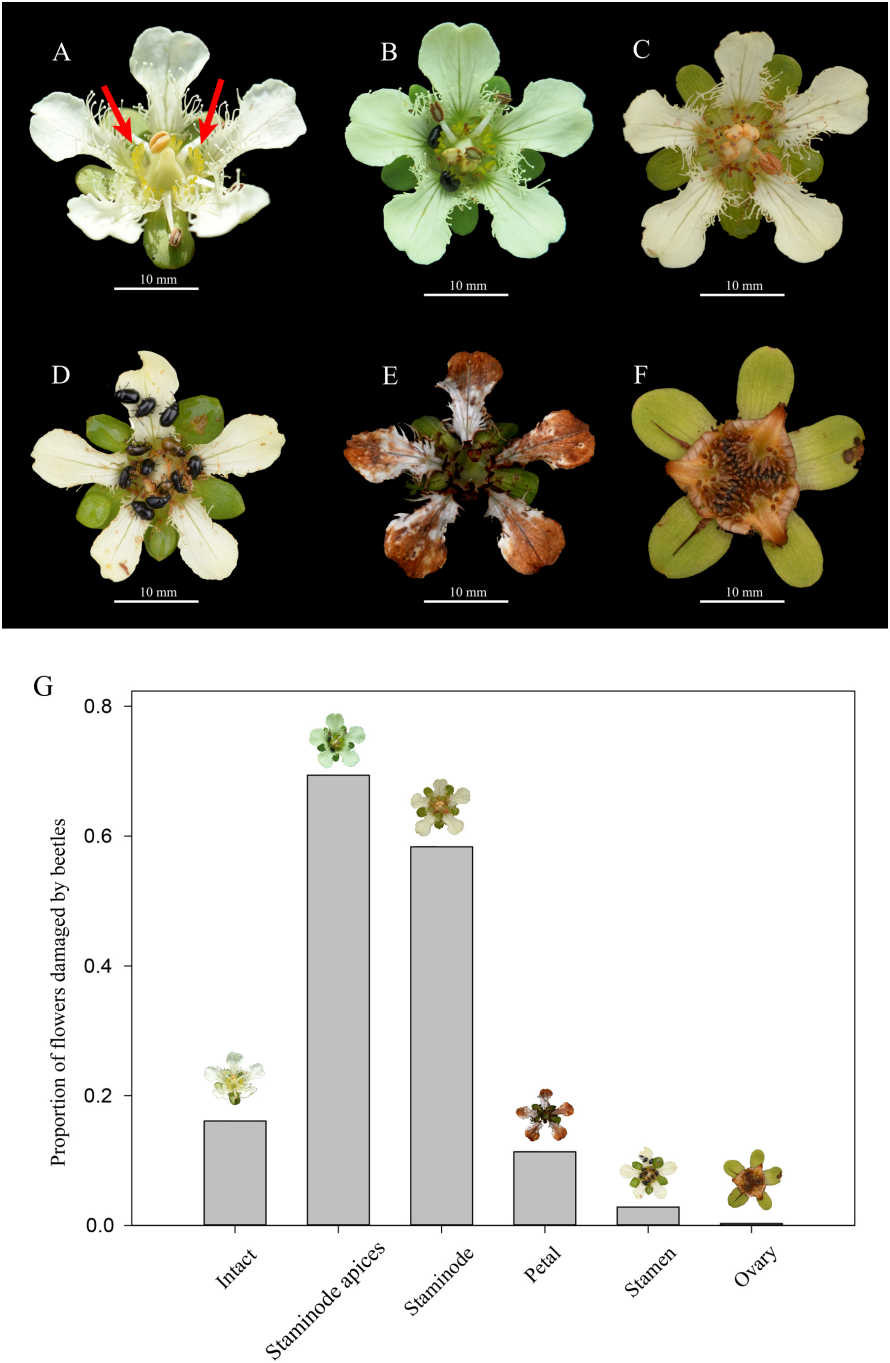
Sequential feeding behavior of beetles on flowers of *Parnassia wightiana*. (A) An intact flower during its the male phase; (B) the staminode apices chewed by two beetles; (C) almost all staminodes have been chewed. (D) stamen chewed by beetles; (E) petal fringes and whole petals chewed by beetles; (F) a fruit without evidence of florivory; (G) proportions of flowers with different floral parts damaged by beetles. Arrow indicates staminode.

The most frequent florivorous beetle observed and collected was *Nonarthra variabilis* (Alticinae; Chrysomelidae). Both males and females foraged in the flowers of *P. wightiana*. These beetles were usually found clinging to staminodes with 1–13 beetles on a flower in the male (anther dehiscence) phase flower. The number of beetles on a female phase flowers was 1–6. *Nonarthra variablilis* selectively and sequentially fed on flower parts, consuming staminode apices first. Then they ate whole staminodes, followed by the fringes ornamented margins of the petals, anthers, and whole petals (Figure 3A–F). A few ate sepals. They were also found feeding on leaves and stems but we never observed them eating stigmas or ovaries (Figure 3E). One beetle was observed feeding on the same flower for > 19 min (*n* = 18 beetles under observation), but none were observed contacting stigma lobes or carrying conspicuous loads of pollen grains. We rarely saw other insects, belonging to other orders, visiting the flowers occupied by beetles. These prospective pollinators also avoided the same flowers with or even after the beetles left.

In Site A, the staminode apices of about 70% of flowers examined showed beetle grazing and 58% of the flowers lacked staminodes, chewed down by beetles (Figure 3G).

### 3.3 Feeding Choice Experiments

For *P. wightiana*, beetles preferred to feed on staminodes instead of whole petals and ovaries (Binomial test: *p* < 0.001). Instead, beetles showed no significant choice bias for staminodes when offered a choice of staminodes and petal fringes (*p* > 0.05), and staminodes and whole stamens (*p* > 0.05). Beetles chose petals against ovaries (*p* < 0.05) and petals against stamens (*p* < 0.01; Figure 2B).

**FIGURE 4:**
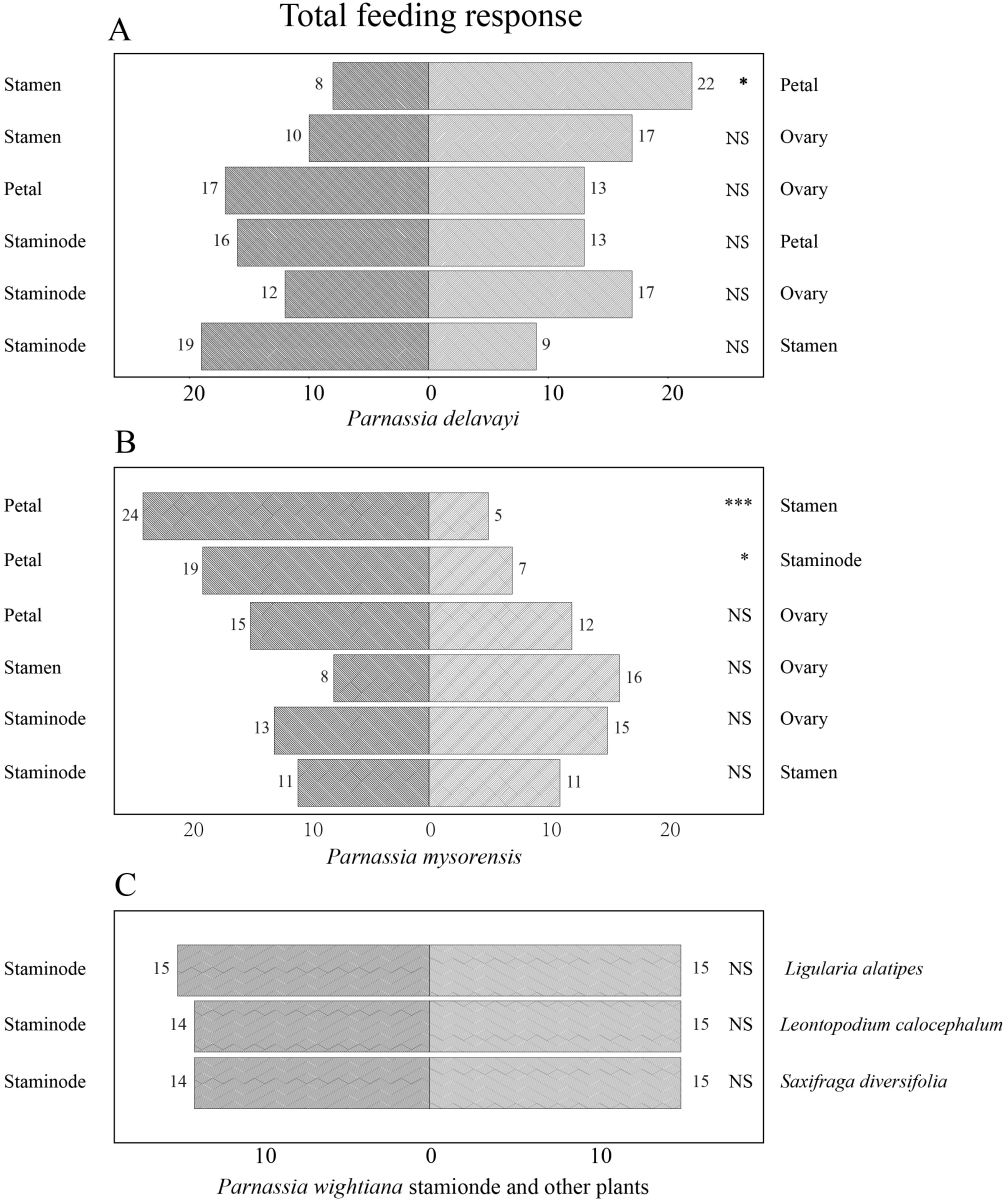
Beetle feeding choice for co‐occurring *Parnassia* and other species. Feeding choices of beetles between floral organs of *Parnassia delavayi* (A) and (B) *Parnassia mysorensis*. (C) Feeding choices of beetles among staminodes of *Parnassia wightiana* and stamens of other co‐occurring plant species including *Ligularia alatipes*, *Leontopodium calocephalum*, and *Saxifraga diversifolia*. NS *p* > 0.05, *p < 0.05, ***p < 0.001.

Beetles did not show a feeding preference for staminodes of *P. delavayi* compared with other floral parts (Figure 4A). For *P. mysorensis*, beetles chose staminodes (*p* < 0.05) or stamens (*p* < 0.001; Figure 4B) over petals. There was no choice bias when beetles were served the staminodes of *P. wightiana* and the stamens of the three co‐blooming plant species (*p* > 0.05; Figure 4C).

### 3.4 Reproductive Success Among Populations

**FIGURE 5:**
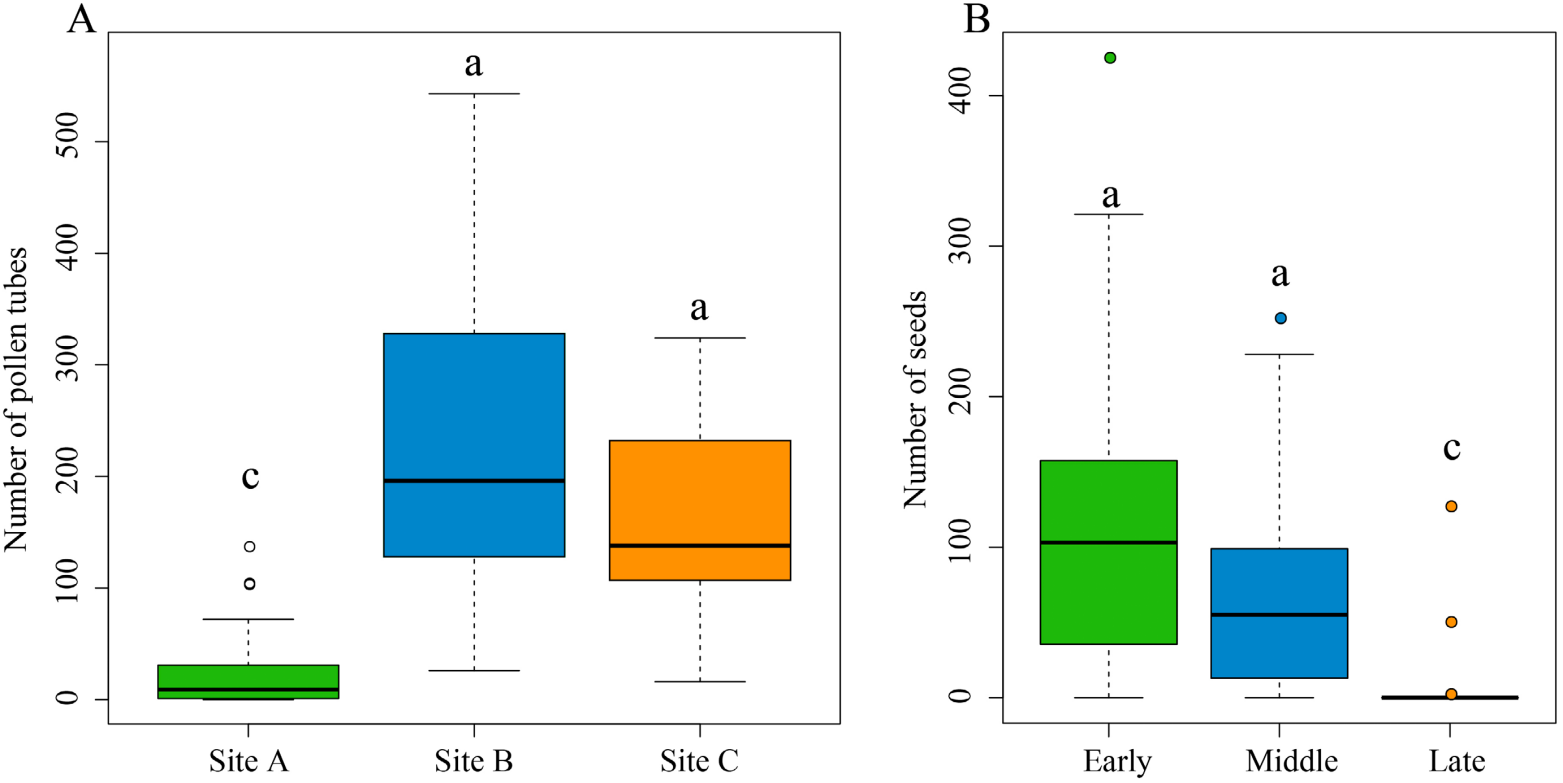
Comparison of reproductive success among populations and flowering periods. (A) Reproductive success measured by the number of pollen tubes in the styles at three sites. (B) Number of seeds/fruit for plants during the early, middle, and late flowering season in Site A. Different lowercase letters indicate significant differences between treatments (*p* < 0.001).

Using the numbers of pollen tubes in styles as proxies for reproductive success, we found that plant reproduction was significantly lower for plants in Site A than plants in Site B and C (*χ* ^2^ = 57.045, df = 2, *p* < 0.001; Figure 5A).

In Site A, where we observed the greatest numbers of beetles in flowers, the number of seeds per fruit decreased over the flowering season (*χ* ^2^ = 41.215, df = 2, *p* < 0.001; Figure 5B). The earlier‐flowering plants had the highest number of seeds/fruit. The later‐flowering plants bearing the most damaged flowers produced the lowest number of seeds/fruit (Figure 5B).

## 4 Discussion

### 4.1 Benefits of the Staminode Whorl

Results of applying field observations and manipulative experiments suggest that the staminode whorl in *P. wightiana* has multiple functions. It attracts prospective pollinators and modulates their behaviors within the flower. Removal of this whorl and/or staminode apices results in a decrease in pollinator visits and shortens the time in which a legitimate pollinator remains in the flower contacting dehiscent anthers or receptive stigmatic surfaces. Without this whorl and/or its apices seed production declines. With the staminode whorl in place and bearing its apical structures, prospective pollen vectors are more likely to contact reproductive organs increasing pollination efficacy (Freitas 2013). However, one limitation of our staminode removal experiment is that we do not know if the decline of floral visitors to staminode removal flowers is due to wounding effect on the change of floral scent distracting floral visitors. Although our human nose cannot detect anything from staminode‐clipped flowers, we cannot exclude the possibility of insects detecting such potential change.

Our feeding choice experiments find that florivorous beetles do prefer staminodes over some of other floral organs, suggesting staminodes are one of cues to attract florivores. However, florivorous beetles have no feeding choice between staminodes and stamens and petal marginal fringes, suggesting that two later organs may also attract beetles. Anyway, the attraction of both pollinators and herbivores to the staminode whorl poses a dilemma for *P. wightiana* as it may do so in other angiosperms, in general (Chautá et al. 2022; Kessler and Chautá 2020). Although intense and general florivory decreases plant fitness (Herrera et al. 2002; Boaventura et al. 2022), selective florivory appears to spare whole pistils. As florivorous beetles prefer staminodes over ovaries, we suggest that staminodes distract the florivores from the ovaries. While staminodes are consumed by beetles in flowers of *P. wightiana*, the sacrifice of many staminodes in a population may ultimately prevent a complete and annual loss of seed output (Maldonado et al. 2015).

### 4.2 *Parnassia* and Multiple Staminode Adaptations in Angiosperms

The role of staminodes in pollination mechanisms has been summarized in some lineages in which these organs are recurrent, fixed in number and genetically proscribed (Decraene and Smets 2001; Endress and Matthews 2006; Chen et al. 2015). Some produce edible rewards including food bodies, nectar or sterile “feeder” pollen (Bernhardt 1996; Bernhardt and Thien 1987). Others accumulate and store rewards produced by other organs. More commonly, they offer visual or olfactory signals to attract pollinators and/or trap, redirect or reposition pollen vectors (e.g., some orchid genera, see Burns‐Balogh and Bernhardt 1985; Edens‐Meier, Pemberton, and Bernhardt 2014). In the genus *Parnassia*, staminode apices mimic nectar irrespective of the real amount of nectar present at their bases, and such signal can remain optically attractive to certain insects for the entire flowering period. This is obvious in our study as manipulated flowers receiving partial or complete removal of staminodes received fewer and shorter visits from pollinators resulting in reduced seed production. As each staminode is a 3‐dimensional advertisement directing foragers to nectar we expected that their complete excision meant that insects could no longer discern the location of the reward.

Furthermore, our experiment also showed that insects lost interest in the flower following removal of the glossy staminode apices producing long‐term constraints in future seed production, highlighting their importance as visual attractants according to Lunau et al. (2020). Others have suggested that it is possible that glossiness, in itself, may also work to deter some antagonists to plant organs (Henríquez‐Piskulich et al. 2023) opening new avenues to understand its meaning especially in flowers (van der Kooi and Spaethe 2023). By analogy to what happens with animals, if the glossy staminode apices could work exclusively to deter beetles we should not find a reduction in pollinator visitation rates and duration as we found when only the apices were removed but nectar was maintained at the staminodes' bases. Instead, we suggest here that glossiness has a specific attractive function in the nectar mimicry systems of *Parnassia* species enhancing its simulation of droplets from above (Lunau et al. 2020), as suggested for flowers in general (van der Kooi et al. 2017). However, in *Parnassia*, and in orchids, e.g., *Ophrys* with glossy speculum (old word for mirror) that mimics the wings of the female insect (Vignolini et al. 2012), it is far more likely that glossiness is a cue for pollinators to land and go about either foraging or sexual behavior.

As in previous studies on other angiosperm species, our observation revealed that the whorl of staminodes may also function as a structure to guide and restrict (modulate) the position and behavior of its pollinators. Since *Parnassia* is protandrous and anthers and stigmas are so close to each other, it is very important for the flower to guide the pollinators to a suitable place to ensure both pollen removal and deposition. In order to reach the real nectar, flower visitor need to land and move to the flower center. Once they are at the floral center the insect extends its proboscis towards the apices of the staminodes, and later to take nectar at the bases of the every staminode heading outside of flower. The visitor needs then to move sequentially around, probing every staminode base without leaving the flower center. When doing that movement, visitors recurrently touched anther and stigma with its abdomen resulting in pollination (S.‐J.Wen et al. unpublished data). Therefore, it is important that the staminode present an attractive structure for floral visitor probing in the male and female phase and thus elicit identical behavior in both flowering phases.

It is suggested that the attractive function of the UV‐reflecting staminode apices is structural and not based on pigmentation (Lunau et al. 2020). We suspect that such a signal can last a long time matching with the long floral lifespan of this species. *Parnassia* produces an open, bowl‐shaped flower, and its nectar can be diluted by rain and mist during the monsoon season in the Hengduan mountains. Therefore, we suspect that the false nectaries of the staminodal apices may give bees and flies a false advertisement that the nectar is always copious and nutrient‐rich. The staminodes are offering a false “super stimulus” (see Edens‐Meier, Pemberton, and Bernhardt 2014) even when the flower is offering little nectar. It will be interesting to investigate the dynamics of nectar production to test the link between nectar mimic signal and true nectar secretion (e.g., honest signaling). If the nectar mimic is a reliable signal to maintain a prospective pollinator's visitation, then we should expect a minimum production of floral nectar to save energy, as floral nectar production is believed to be costly for plants (Pyke and Ren 2023).

Our manipulative experiment suggests that these glossy apices are also preferred by herbivorous beetles. However, the feeding behavior of our beetles followed an almost stereotyped order of preference in which apices are preferred to the whole staminodes. Whole staminodes, in turn, are more likely to be consumed next followed by the thin, fringed petal margins, whole petals and finally, the stamens. They show little interest in the ovaries. There are probably several reasons why these insects divide the flower into courses as if at a banquet. First, the glossy apices obviously provide the original cue that attracts beetles to the flower as they reflect UV, contrasting with other floral organs (but see van der Kooi and Spaethe 2023 for the misguiding function of glossiness). Second, while the apices do not function as nutrient sources for nectar‐drinking pollinators, it is obvious that herbivorous beetles, with biting mandibles, have no problem consuming these mimics. What follows, probably reflects a gradation of nutritional contents and plant tissue reinforcement. Note that, after eating the staminodes the beetles move on to the thin petal margins and not to the thicker, vascularized stamens. One would think that the stamens would be next as pollen grains should contain more amino acids and lipids for beetles provisioning their eggs. Perhaps these chrysomelids lack mandible and/or gastro‐intestinal modifications to rupture exines (Bernhardt 1996). There may be a second reason for their initial preference for staminodes. In some plants pigmented floral organs are toxic to herbivores (Gronquist et al. 2001). Should we also presume that the young, greenish pistil is left unmolested because its ovary walls contain more indigestible sclereids, or lignin, or crystals compared to the other floral organs? Future sectioning and selective staining of these structures will answer the question.

### 4.3 Addressing the Functional Dilemma

An attraction to herbivores poses a functional dilemma to plants, while the same attraction to pollinators ensures reproductive success (Herrera et al. 2002; Boaventura et al. 2022). The effect of beetle florivory on plant reproduction is twofold. First, beetle‐damaged flowers receive fewer visitations from members of local pollinator guilds. Indeed, prospective pollinators rarely visit flowers still occupied by beetles. High beetle infestations at Site A correlate with lower seed production. Second, damage could lead directly to fruit losses in pollinated flowers as we observed some beetles biting into the floral stem and that may impede or stop fruit development. In contrast, flowers blooming before beetle populations peaked had higher levels of seed production.

We do not interpret the whorl of staminodes as functioning as a dependable and sacrificial investment ensuring that beetles, once satiated, will now leave the pistil unmolested. This would be a possibility only if the act of pollination occurred before beetles arrived, but this is a protandrous flower. In our populations, beetles are already feeding on the male phase flowers days before the stigma becomes receptive.

Is there a solution to the attraction dilemma in this species? Early flowering may be one possible and partial response to the indirect effects of beetles on reproductive success. A systematic survey of flower‐visiting beetles at our sites by Li, Ren, and Li (2021) suggested that many species of flower‐visiting beetles are specific to certain microhabitats and/or show limited periods of seasonal activity. This is confirmed by the restricted activity of *N. vaiablilis*. It was observed most often in wet meadows at elevations of 3300 m. These insects did not emerge in force to attack flowers of *P. wightiana* until mid‐July. At a higher elevation, the populations of *P. wightiana* were flowering already in June. Early bloomers escape predation earning a higher level of reproductive success. It is unusual that plant populations at a higher elevation, growing under a regime of lower temperatures, should flower before populations of the same species at a lower elevation (Z.‐X. Ren, unpublished data). We suggest this is, in part, an example of a directional selective response to herbivory. Of course, intrapsecific variation in floral phenology may be caused by other environmental factors, and we expect such prospects to open tantalizing opportunities for further studies on montane populations of flowering plants, their pollinators and herbivores.

## Author Contributions

**Shi‐Jia Wen:** data curation (equal), formal analysis (equal), investigation (equal), writing – original draft (equal). **Shan Chen:** data curation (equal), formal analysis (equal), investigation (equal). **Andre Rodrigo Rech:** methodology (equal), visualization (equal), writing – review and editing (equal). **Ling Ji:** data curation (equal), visualization (equal), writing – review and editing (equal). **Hong Wang:** conceptualization (equal), project administration (equal), supervision (equal), writing – review and editing (equal). **Zhiyong Wang:** funding acquisition (equal), supervision (equal), writing – review and editing (equal). **Ding Wu:** conceptualization (equal), funding acquisition (equal), supervision (equal), writing – review and editing (equal). **Zong‐Xin Ren:** conceptualization (equal), data curation (equal), funding acquisition (equal), methodology (equal), project administration (equal), writing – original draft (equal), writing – review and editing (equal).

## Conflicts of Interest

The authors declare no conflicts of interest.

## Supporting information


**Figure S1.** Beetle chewing peduncles of bagged (A) and marked (B) flowers.

## Data Availability

Full dataset was deposited in the Dryad Data Repository: https://doi.org/10.5061/dryad.b2rbnzsqg. URL: https://datadryad.org/stash/share/1FkcmTRuyLmk4mA2Ljif3DaNmX1wK0Q0ifhSLC6imO8.
