# Supplementary figures and images for: The Functional Dilemma of Nectar Mimic Staminodes in Parnassia wightiana (Celastraceae): Attracting Pollinators and Florivorous Beetles
Source: Ecol Evol. 2024 Sep 30;14(10):e70380. doi: 10.1002/ece3.70380 (PMC11442331; doi:10.1002/ece3.70380)

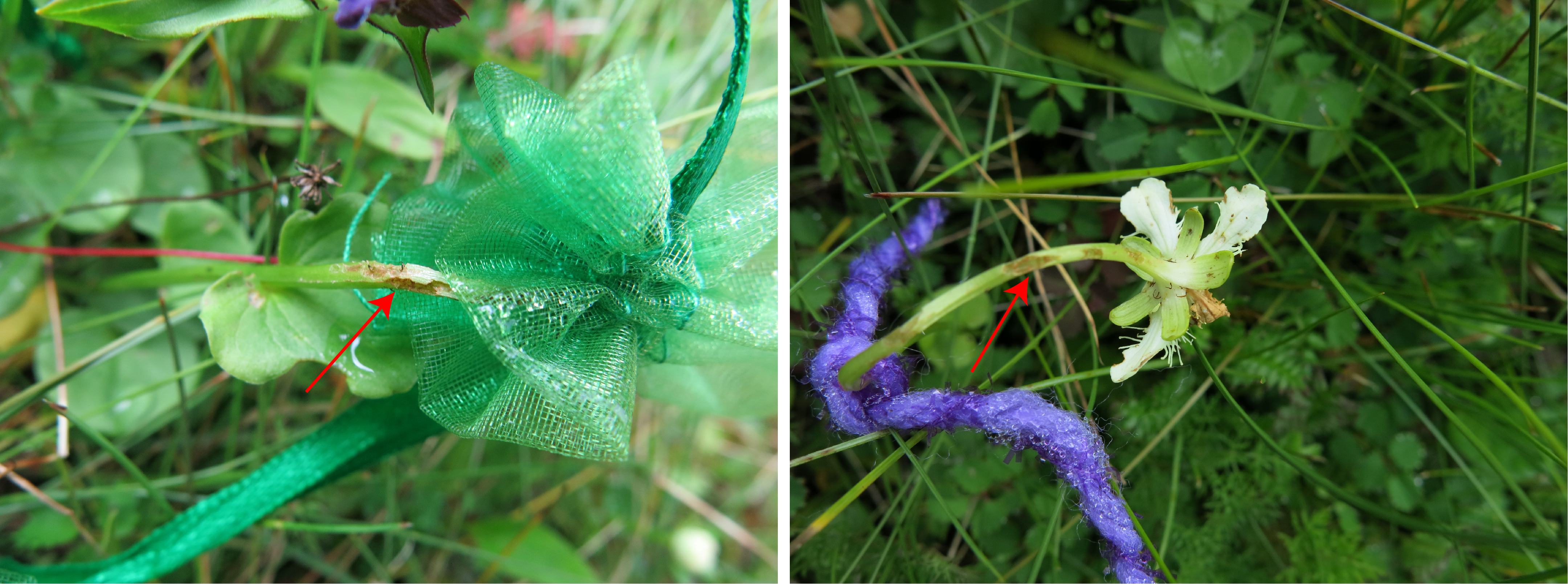

Supplement: Supplementary file 1 — Figure S1. Beetle chewing peduncles of bagged (A) and marked (B) flowers. [file ECE3-14-e70380-s001.jpg]
